# Targeting protein dynamics in drug design

**DOI:** 10.1186/1758-2946-5-S1-O1

**Published:** 2013-03-22

**Authors:** Rebecca C Wade

**Affiliations:** 1 Molecular and Cellular Modeling Group, Heidelberg Institute for Theoretical Studies (HITS), Germany; 2 Center for Molecular Biology (ZMBH), Heidelberg University, Heidelberg, Germany

## 

The dynamic nature of protein structures provides challenges and opportunities for ligand design. I will first discuss some of the different ways in which protein dynamics can be targeted, giving examples from our experience in the design of inhibitors of thymidylate synthase [1-3]. I will then describe the development of a computational toolbox (TRAPP: TRAnsient Pockets in Proteins) to identify and visualize transient pockets in proteins that may be exploited in ligand design.
